# Spatial mapping of lichen specialized metabolites using LDI-MSI: chemical ecology issues for *Ophioparma ventosa*

**DOI:** 10.1038/srep37807

**Published:** 2016-11-24

**Authors:** Pierre Le Pogam, Béatrice Legouin, Audrey Geairon, Hélène Rogniaux, Françoise Lohézic-Le Dévéhat, Walter Obermayer, Joël Boustie, Anne-Cécile Le Lamer

**Affiliations:** 1 Université Rennes 1, UMR CNRS 6226 PNSCM, 2 Avenue du Pr. L. Bernard, 35043 Cedex, France; 2 Institut d’Électronique et de Télécommunications de Rennes, Université Rennes 1, UMR CNRS 6164, 263 Avenue du Général Leclerc, 35042 Cedex, France; 3 INRA, UR 1268 Biopolymers Interactions Assemblies F-44316 Nantes, France; 4 Universitat Graz, Institut Karl Franzens, Holteigasse 6, A-8010 Graz, Austria; 5 Université Toulouse 3 Paul Sabatier, UFR Pharmacie, 118 Route de Narbonne, 31062 Toulouse, France.

## Abstract

Imaging mass spectrometry techniques have become a powerful strategy to assess the spatial distribution of metabolites in biological systems. Based on auto-ionisability of lichen metabolites using LDI-MS, we herein image the distribution of major secondary metabolites (specialized metabolites) from the lichen *Ophioparma ventosa* by LDI-MSI (Mass Spectrometry Imaging). Such technologies offer tremendous opportunities to discuss the role of natural products through spatial mapping, their distribution patterns being consistent with previous chemical ecology reports. A special attention was dedicated to miriquidic acid, an unexpected molecule we first reported in *Ophioparma ventosa*. The analytical strategy presented herein offers new perspectives to access the sharp distribution of lichen metabolites from regular razor blade-sectioned slices.

Lichens are a world-widespread consortium of fungal and photosynthetic partners. The high specialization in lichen tissue metabolism leads to diverse metabolite distribution corresponding to secondary metabolites, preferably named specialized metabolites. For example, lichens tend to allocate their most efficient grazing-deterrent compounds to their reproductive parts that are the most valuable for lichen fitness, in agreement with the optimal defense theory^1^. The discovery of such distribution patterns most often relies on extraction of targeted tissues for chemical analyses, sometimes guided by specific features of the analytes such as UV fluorescence^2^. Many lichen compounds can also be stained by exogenously applied chemicals (spot tests on cortex, medulla or apothecium). Both approaches appear limited since sharp details of distribution are lost when analyzing bulk tissues, while techniques based on functional group histochemistry do not distinguish between individual compounds and lead to distortions of localization. MSI techniques underwent significant technological improvements during the last decade so that they gained considerable importance in the field of plant metabolites imaging, the most prevalent method being MALDI-MSI (Matrix Assisted Laser Desorption and Ionization)^3^. We first reported on auto-ionisability of all main classes of lichenic compounds using matrix-free LDI-MS and emphasized the potential of this technique as a blitz-screening compatible dereplication tool^4^. Indeed, lichen analytes did not require a chemical matrix for ionization avoiding the tricky step of matrix spraying and the interference between the high matrix ion background in the low mass range and sample metabolites. Lack of matrix is also a tremendous advantage in the field of imaging since applying MALDI matrices complicates sample preparation for imaging and might disturb the native distribution of the studied metabolites^5^. *Ophioparma ventosa* (L.) Norman., also referred to as the Alpine bloodspot owing to its blood-red fruiting bodies (apothecia) and a grayish thallus, represents a well-fitted model to investigate the tissue-specific accumulation of lichen metabolites. Preliminary DART-MS experiments emphasized specific distribution patterns of *O. ventosa* specialized metabolites between the apothecia and the thallus and within the depth of its thick thallus paving the way for imaging mass spectrometric analyses on this lichen^6^. The structural diversity of specialized metabolites described in this lichen (Figure S1)^7^ also makes it a relevant model to demonstrate the broader applicability of LDI-MSI in the wide field of lichenology. Besides, the material investigated here was of specific interest as it contained miriquidic acid, a rare depside which was solely described for this species on this particular sample collected in Austrian Alps^6^. Given the aggressiveness of *O. ventosa* and its trend to overrun neighbouring lichens, an hypothesis to explain the arising of this additional molecule is that it might be acquired from overgrown lichens rather than being biosynthesized by *O. ventosa* itself, as previously suggested for other additional metabolites^7^,^8^. In our specific case, locally occuring miriquidic acid-producing lichen species represent likely candidates to account for the presence of this depside within our sample (e.g *Miriquidica garovaglii*)^9^. It was therefore worth checking (i) how miriquidic acid was distributed in a piece of thallus and (ii) whether miriquidic acid was ascribed to the basal layers of the lichen, as expected if acquired from overgrown lichens. In our study, we intended to image the distribution pattern of specialized metabolites from *O. ventosa* with a specific insight into miriquidic acid. LDI-MSI could establish the spatial mapping of all specialized metabolites known from our sample with a spatial resolution of 50 μm including miriquidic acid and even one of the trace pigments recently described within the apothecia of *O. ventosa* (Figure S1)^10^. Their distribution patterns are consistent with the ecological roles previously proposed for these metabolites.

## Results

### Lichen material and preparation of the slices

**Figure 1:**
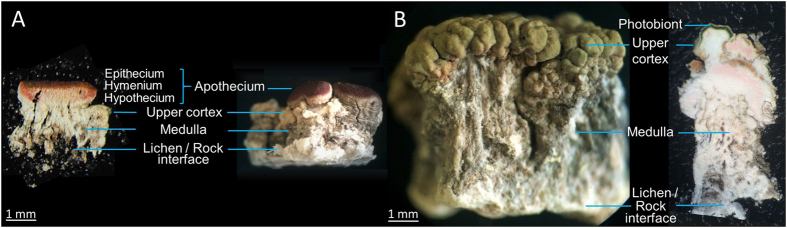
Lateral macroscopic views of *O. ventosa* from both sampling sites revealing the different anatomical features alongside a cryosectioned piece of an apotheciate thallus (Tyrol sample) (**A**) and a hand-cut piece of a non-apotheciate thallus (Styria sample) (**B**).

*Ophioparma ventosa* appears as a crustose epilithic lichen forming large patches reaching up to 15 cm diameter. Its blood-red fruiting bodies (=apothecia) are delimited by an algae-containing external rim concolour with the rest of the thallus called thallin margin. The appearance of this species is known to be highly variable regarding the color and the thickness of the thallus^8^,^7^. Hence, two different samples from Austrian Alps were considered during the course of this manuscript to take into account the morphological variety of this lichen. The first sample, referred to as Tyrol sample, was collected in the Tyrol state in southwest Austria (Fig. 1A). It exhibits a yellowish colour and a rather thin thallus structure (1–3 mm). The second lichen displaying a greenish colour and a thick thallus (3–10 mm) was collected in the state of Styria, in the southeast of Austria (Styria sample) (Fig. 1B).

The histological structures of slices containing and lacking apothecia obtained from both lichen samples are displayed in Fig. 1. The cross section of an apothecium reveals the hymenium which represents the spore-bearing layer of the fruiting body. The epithecium is the red tissue present at the surface of the hymenium layer, formed by the branching of the ends of the paraphyses above the asci. Conversely, the hypothecium refers to the hyphal layer beneath the hymenium in an apothecium. The hypothecium of *O. ventosa* is faint pink, especially in its basal parts^11^. *Ophioparma ventosa* develops an internally-stratified thallus typical of the so-called heteromerous lichens divided into three main layers that are upper cortex, photobiont layer and medulla. The algal cells, in the present case belonging to the *Trebouxia* genus, are arranged in a discrete layer immediately below the upper cortex. Due to the areolate structure of the thallus, the algae trailing the upper cortex might arise in an anticlinal orientation. The thickest lichen layer, named medulla, is the histological layer present below the photobiont to the surface of the rock. As a crustose lichen, *O. ventosa* lacks a lower cortex. Its fungal filaments extend downwards into the rock substrate from its entire surface, forming an hyphal layer inside the rock. The layer could be 7–12 times as thick as the thallus present at the surface of the rock^12^. Therefore, the lichen cannot be removed from its substratum without being damaged which accounts for the poor integrity of the lower parts of the thallus, which might further crumble upon slicing.

Two different slicing procedures were applied to the lichen material for samples of both locations: cryosectioning (Fig. 1A) and hand-cutting (Fig. 1B). For the cryosectioning procedure, the use of organic solvents was avoided to circumvent de-localization of hydrophobic metabolites of *O. ventosa* and 40 *μ*m thick transverse sections were cut from frozen pieces of thallus resulting in a significant crumbling of the medulla. Hand-cutting merely used a razor blade, as regularly performed by lichenologists, to afford undamaged slices with a thickness exceeding 100 *μ*m.

### Chemical investigation of micro-samples of *O. ventosa*

**Figure 2:**
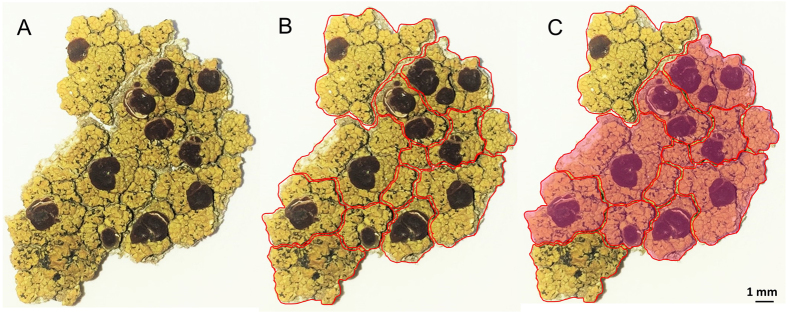
Longitudinal distribution of miriquidic acid in a piece of *Ophioparma ventosa* thallus (Tyrol sample). Piece of thallus (**A**). Division of a piece of thallus in small fragments (**B**). Rose patches refer to areas containing miriquidic acid, as revealed by TLC monitoring (**C**).

To assess whether miriquidic acid was evenly distributed in the thallus, we first checked the chemical profile of 50 random pieces of thallus from the Styria sample. Each fragment was cut into upper and lower halves to monitor their chemical content by Thin Layer Chromatography (TLC) (Figure S2). These micro-analyses revealed that miriquidic acid occurs in only two of them, and when it is present, it seems confined to the lower part of the medulla. Then, the distribution of miriquidic acid was studied in the specific context of a piece of thallus taken from the Tyrol sample. For this purpose, several pieces of lichen were divided into small fragments in accordance with the areolate structure of the thallus as depicted in Fig. 2B and S3. TLC monitoring of each fragment confirmed that miriquidic acid did not occur in every fragment and it is noteworthy that the lichen fragments containing this depside appeared contiguous to one another, defining patches as displayed in Fig. 2C and S3. Once again, miriquidic acid was ascribed to the lower half of the thallus pieces. Hence, these results indicate an uneven longitudinal distribution of miriquidic acid and allowed us to select pieces of lichen containing or lacking miriquidic acid for LDI-MSI experiments.

### 355 nm-UV Laser desorption/ionization time of flight (LDI-TOF) detection of *O. ventosa* specialized metabolites

We recently outlined that lichen metabolites ionized under UV irradiation with a conventional nitrogen laser (337 nm) fitted to the MALDI instrument^4^. Since the present study uses a spectrometer with a 355 nm smartbeam laser, a dichloromethane extract of *Ophioparma ventosa* was analyzed as a control to check if compounds could still be ionized at this different wavelength without matrix assistance. This preliminary analysis revealed that a 355 nm laser triggers a satisfying ionization of all main compounds encountered within our *O. ventosa* sample, with comparable fragmentation patterns. Signals from this mass spectrum were thus assigned by comparison to prior 337 nm – LDI analysis^4^. Names and structures of specialized metabolites described from *O. ventosa* are given in Figure S1.

**Figure 3:**
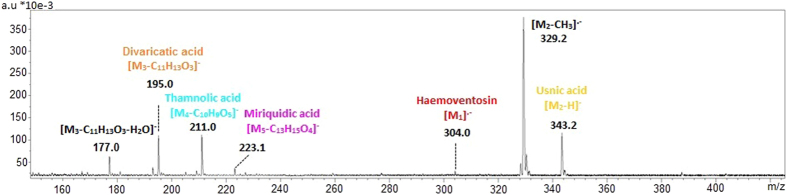
Negative-ion mode LDI mass spectrum of a dichloromethane extract of *Ophioparma ventosa* displaying ions selected for imaging mass spectrometry with related colors.

Deprotonated usnic acid (*m/z* 343) and the molecular ion of haemoventosin (*m/z* 304) were selected to image the distribution of these metabolites. Depsides underwent a significant fragmentation under 355 nm laser desorption ionization similar to that triggered at 337 nm so that divaricatic, miriquidic and thamnolic acids were better detected through their fragments than their deprotonated molecule. Depsides were consequently imaged by their alcohol moieties, released through breakage of their ester bond as previously reported (divaricatic acid: *m/z* 195, miriquidic acid: *m/z* 223 and thamnolic acid: *m/z* 211)^4^ (Fig. 3).

### LDI-MSI of *O. ventosa* specialized metabolites

**Figure 4:**
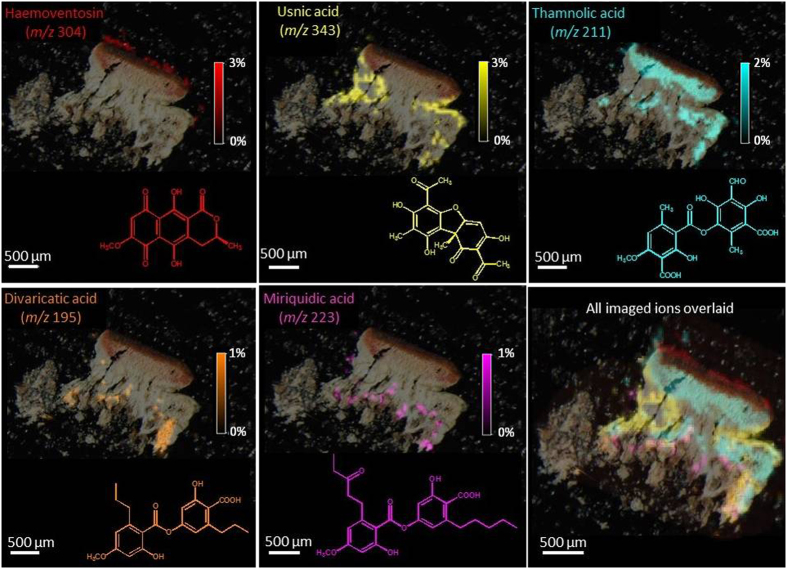
From left to right: Molecular images of haemoventosin, usnic acid, divaricatic acid, thamnolic acid, miriquidic acid and all overlaid ions in a cryosectioned piece of an apotheciate piece of thallus from the Tyrol sample of *Ophioparma ventosa*. Intensities of ions in the imaged spots are color coded using a heat map with relative intensities given as indicated on the color scale bars.

**Figure 5:**
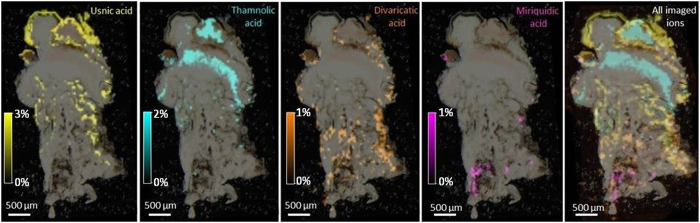
From left to right: Molecular images of usnic acid, divaricatic acid, thamnolic acid, miriquidic acid and all overlaid ions in a hand-cut piece of an apotheciate piece of thallus from the Styria sample of *Ophioparma ventosa*. Intensities of ions in the imaged spots are color coded using a heat map with relative intensities given as indicated on the color scale bars.

*In situ* assessment of *O. ventosa* specialized metabolites is displayed on two series of mass spectrometric images from the two sampling sites (Tyrol: Fig. 4 and S4 and Styria: Fig. 5 and S5). A first outcome is that LDI-MSI afforded ion images of similar qualities from both 40 *μ*m thick cryosectioned slices and *ca.* 100 *μ*m hand-cut sections. Then, the distribution patterns retrieved from the lichens of both sampling sites and from either apotheciate or non-apotheciate pieces of thallus are similar. Specialized metabolites could be imaged with a resolution of 50 *μ*m, showing organized and specific spatial allocations for each compound, which do not overlay.

**Figure 6:**
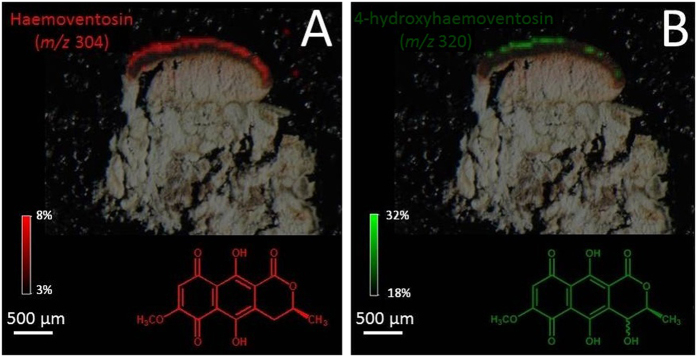
Molecular images of naphthazarine pigments in the Tyrol sample of *Ophioparma ventosa* (**A**): haemoventosin *m/z* 304 and (**B**): 4-hydroxyhaemoventosin *m/z 320*). Intensities of ions in the imaged spots are color coded using a heat map with relative intensities given as indicated on the color scale bars.

Haemoventosin was localized in the red epihymenial layer. While this molecule remains the major pigment of the fruiting bodies, we recently isolated a variety of minor pyranonaphthoquinones from the apothecia of *O. ventosa*^10^. Their trace amounts limited their imaging but 4-hydroxyhaemoventosin could be ascribed to the red epihymenial layer, thus being co-localized with haemoventosin (Fig. 6). Usnic acid is densely distributed above the *Trebouxia* photobiont layer and along internal furrows. Thamnolic acid is confined to the hypothecium and upper medulla parts of the lichen, associated with faint pink-colored patches. At last, divaricatic and miriquidic acids are allocated to the lower medulla of the lichen extending downwards to the lichen/rock interface. Molecular images acquired from samples not displaying miriquidic acid in both Tyrol and Styria lichens are presented in Figures S4 and S5.

Overall, these results were in full agreement with those provided by DART-MS which ascribed haemoventosin, usnic and thamnolic acids to the upper part of the thallus whereas divaricatic acid was rather detected from the lower half of the lichen^6^. Non-detection of miriquidic acid from the previous DART-MS investigated pieces of thallus might refer to the uneven allocation of this depside.

## Discussion

LDI-MSI appears as a versatile approach for lichen metabolites mapping. Regarding the thickness of the slices, regular slices obtained using a razor blade are perfectly suitable for imaging purposes, bypassing the mandatory need for expensive slicing facilities. Haemoventosin, usnic acid,. divaricatic acid and thamnolic acid are diagnostic compounds of *Ophioparma ventosa*^8^,^7^. Their occurrence in each slice supports their taxonomic value, and their organized, non-overlapping and constant distribution patterns suggests a specific ecological relevance for each of them.

As expected, pigments such as haemoventosin and its minor derivative 4-hydroxyhaemoventosin are confined to the apothecia, especially to the epithecium. The reproductive structures are often reported to produce specific lichen compounds or known to be more concentrated in phenols than the remaining thallus^13^, in accordance with the optimal defense theory^1^,^14^. Haemoventosin belongs to a structural class (quinones) exerting a wide array of biological activities of ecological significance including antibiotic, antifungal^15^ and cytotoxic properties^10^. Moreover, haemoventosin putatively acts as a UV screening filter to protect spores during their maturation within asci. As haemoventosin forms a film at the surface of asci apices, this quinone most likely spreads on spore surface which might be a beneficial effect for the initial steps of spore germination, by inhibiting the growth of competing microorganisms. Lichens were also found to host substantial communities of non photo-autotrophic bacteria, that are increasingly regarded as integrated partners of the lichen symbiosis^16^. It was presumed that the arising of haemoventosin was linked to the establishment of the atypical bacterial communities of *O. ventosa*, selecting bacterial strains able to withstand such metabolites^16^,^17^.

LDI-MSI revealed usnic acid to be concentrated in cortical areas that contain the photobiont. Through the use of vibrational spectromicroscopy approaches and scanning electron microscopy, comparable distribution patterns were evidenced in different lichens^18^,^19^. In the context of lichen symbiosis, the photobionts display an increased resilience suggesting that a substantial photoprotection is provided by the cortical mycobiont layer above the photobiont. Given the strong UV absorption of usnic acid^20^, its cortical distribution suggests its involvement in photoprotection of the sensitive algal layer^13^,^21^. However, the arising of this phytotoxic mycobiont-derived product^22^,^23^,^24^,^25^ at the surface of the algal partner^26^, or even within it^27^, might appear surprising. Usnic acid was indeed proven to act as an allelochemical exerting phytotoxic effects that are nevertheless higher towards free-living alga than on aposymbiotically grown lichen photobionts. This shall be regarded as an adaptation resulting from long term co-evolution of these algae with fungi that produce toxic specialized metabolites^28^. To maintain a harmonious growth pattern of the thallus, the mycobiont, whose growth capacities are limited, has to regulate the metabolism and the division rate of its photobiont through chemicals^29^,^30^. More widely, usnic acid displays pleiotropic effects including strong antibacterial effects^31^, antifeedant activity, toxicity towards herbivorous insects^32^,^33^ and metal-binding properties under acidic conditions^34^ that might lower availability of toxic ions to photobiont cells^35^. It is worth pointing out that usnic acid also occurs in deeper parts of the lichen thallus, where it defines reticulated structures. This secondary distribution pattern can be understood by referring to the brain-like growth of *O. ventosa* thallus: when new squamules are developed on the surface, the old surface of the lichen (including cortex and algae) gets buried in a self-overgrowing process. The deep furrows of usnic acid might then refer to the former localization of the photobionts.

Thamnolic acid was found to be mainly located in the upper part of the medulla for both samples, but also within hypothecial tissue for apotheciate samples (Fig. 4). A few works previously reported on thamnolic acid distribution within lichen thalli, most often arising in the sub-apothecial tissues as well as in the hypothecial and thalline tissues^36^. Regarding *Ophioparma* species, thamnolic acid is given responsible for the pink color of the basal hypothecium layers (Fig. 1B)^11^,^37^. This depside is found in a wide array of lichens growing on acidic substrata as *O. ventosa* that displays a mid pH range of 4.4^38^. With a pKa_1_ of 2.8, thamnolic acid might represent a selective advantage to cope with increasing acidic air and rain pollution^39^,^40^,^41^. Likewise, one can imagine that thamnolic acid produced by *O. ventosa* might help outcompeting other lichens growing in its environment in acidifying conditions. As an example, the commonly associated *Rhizocarpon geographicum* was proven to be severely damaged by acid rain^42^. Spatial allocation of compounds involved in control of acidity tolerance in lichens is not considered to play a key role in their functioning^41^. However, hypothecium represents a very valuable tissue for lichen fitness. Therefore, the partitioning of thamnolic acid might be regarded as a further example of the optimal defense theory.

LDI-MSI spatial mapping allocated divaricatic acid to the lower parts of the medulla, even at the interface with the rock for the Tyrol sample, thus making divaricatic acid a candidate to account for chemical weathering^43^. Some lichen substances in direct contact with the mineral surfaces of porous rocks were reported to increase mineral dissolution rates and may contribute in the chemical weathering process^43^,^44^,^45^,^46^,^47^. Indeed, in a comparative study of the lichen-rock interface of different epilithic lichens, the mean weathering depth beneath *O. ventosa* was much thicker than with the other lichens^43^. Divaricatic acid was among the first molecules reported to occur in the rock beneath epilithic lichens and the only *O. ventosa* metabolite that has been detected in the weathered rock^43^,^48^. Besides, cation-chelating properties of lichen substances that accumulate on the outer surface of the hyphae might also step in metal ion homeostasis and heavy metal tolerance^49^,^50^,^51^. As such, divaricatic acid was proven to promote Cu^2+^ uptake^52^ by adsorbing this ion. Since Cu stands among the rarest micronutrients, divaricatic acid might be crucial in supplying this cofactor. Promotion of the uptake of metals needed as micronutrients might then broaden the ecological niche of lichens in nutrient-poor habitats^52^.

We first reported on the presence of miriquidic acid in a sample of *O. ventosa* collected in the Tyrol state in Austria^6^. Alongside aforementioned metabolites that are constant in this lichen, a vast array of additional molecules could be occasionally detected including atranorin, stictic, norstictic, psoromic, salazinic, gyrophoric, alectoronic and *α*-collatolic acids^7^. Since lichens belonging to the *Ophioparma* taxa grow aggressively, one current hypothesis for the origin of such additional compounds is that they could be acquired from overgrown lichens. This assumption is further strengthened by the co-occurrence of lichens containing such specialized metabolites in their close environment. As an example, in the course of its study on Finnish *O. ventosa* samples, Skult reported on the “unexpected” presence of atranorin in some of its specimens^8^,^11^, assuming that atranorin might arise from a contamination in the thick prothallus^8^. Likewise, May suggested that the thick thallus of *O. ventosa* might easily hide the remnants of long overgrown lichens to account for the arising of atranorin and additional depsidones in some specimens^7^. As such, the occurrence of host-synthesized substances was recently observed in the lichenicolous lichen *Miriquidica invadens* that accumulates 5-*O*-methylhiascic acid from the parasitized *Sporastatia polyspora*^53^. Regarding our samples, miriquidic acid might stem from overgrown species containing this depside which are described in both sampling sites such as the squamulose *Miriquidica garovaglii*. LDI-MSI analysis revealed that miriquidic acid was located in the basal medullary layer of the thallus. Such a basal distribution appears to be compatible with an acquisition from overgrown lichens. Moreover, analysis of further slices obtained from the same thallus do not reveal the presence of miriquidic acid. This validates the uneven longitudinal distribution highlighted earlier, and therefore supports the acquisition from a parasitized lichen, rather than a new *O. ventosa* chemotype.

LDI-MSI is here first described as a powerful tool for molecular mapping of compounds in lichen sections. From a methodological perspective, some advantageous features are worth being stressed. At first, the lack of matrix facilitates sample preparation and ensures an optimal spatial resolution. Likewise, the versatility of the technique regarding the thickness of the slice can lead to image the distribution of metabolites from regular hand-made slices. This micro-scale mapping enabled to establish the distribution pattern of all the molecules identified from our sample of *O. ventosa* and supported the putative ecological significances of most of them. Imaging of miriquidic acid reveals a distribution pattern compatible with its acquisition through overgrown lichen thalli. Considering such aspects, LDI-MSI approaches might represent new opportunities to decipher molecular strategies underlying dynamism and equilibrium in a saxicolous lichen mosaic. Little is known about aspects conferring advantage and factors promoting stability in multi-species communities but chemistry and allelopathy are presumed to be of paramount importance^54^ as recently proven for endophytes^55^. Therefore, delineating chemical interactions ongoing at marginal contacts between lichen thalli might advance the understanding of such complex relationships.

LDI-MSI techniques might also shed light on poorly known metabolic processes lying at the very heart of these fascinating symbioses. Many aspects of this association yet have to be characterized regarding the exact identity of the transferred metabolites and subsequent fate of the translocated material in fungal tissue^56^. This lack of knowledge most likely stems from difficulties in investigating such metabolic interactions both *in situ* and at high spatial resolution. Addressing these shortcomings, LDI-MSI provides new opportunities for the investigations of many aspects of the lichen symbiosis, including the dynamics of cellular interaction. Imaging mass spectrometry techniques combined with stable isotope labelling can track the spatio-temporal dynamics of metabolic interactions in symbiotic systems^57^. A proof of principle on *Xanthoria parietina*, using pre-labeled photobiont, an unlabeled mycobiont and a reconstituted lichen by co-cultivation of the two partners, indicated the transfer and usage of algal metabolites for biosynthetic purposes by the mycobiont^58^. Altogether, this study highlights the interest of LDI-imaging mass spectrometry as a simple and versatile strategy to assess the distribution of metabolites within lichens and more widely paves the way for its future application to polyphenolic structures in the wider realm of natural products. The narrow correlation between the localization of the compounds and their presumed ecological significance demonstrates the relevance of LDI-MSI as a privileged tool for chemical ecology studies.

## Methods

### Lichen material

Two samples of *Ophioparma ventosa* of different thicknesses were harvested: a first sample was collected in Tyrol (Austria), 500 meters south of Obergurgl (elevation 1800–1850 m). The lichen was collected and identified by one of the authors (J.B.) in 04/2009. A voucher specimen was deposited at the herbarium of laboratory PNSCM with the reference JB/09/158. This sample is referred to as Tyrol. Sample 2 was harvested in Styria (Austria) at the south of the lake Grosser Winterleitensee (elevation 1950–2000 m). The specimen was collected and determined by one of the authors (W.O.) in 09/2014. A voucher specimen (No. 13218a) was deposited at the herbarium GZU. In the rest of the manuscript, this sample is identified as Styria.

### Sample preparation for LDI mass spectrometry imaging

Fourty microns-thick transverse frozen sections were cut using a cryostat (Leica, Milton Keynes, UK) and fixed on a carbon-conductive adhesive tape which was in turn fixed on an indium tin oxide (ITO) slide (Bruker Daltonics, Bremen, Germany, cat no 237001).

All MSI measurements were performed using an Autoflex-Speed MALDI-TOF/TOF spectrometer (Bruker Daltonics, Bremen, Germany) equipped with a Smartbeam laser (355 nm, 1000 Hz) and controlled using the Flex Control 3.4 software package. The mass spectrometer was operated with a negative polarity in the reflectron mode. Spectra were acquired in the mass range of m/z 100–600 for all (x, y) coordinates corresponding to the imaged tissue.

The laser raster size was set at 50 microns. The signal was initially optimized by manually adjusting the laser power and the number of laser shots fired. Accordingly, full-scan MS experiments were run by accumulating 400 satisfactory laser shots per raster position, and using the laser power leading to the best signal-to-noise ratio. Image acquisition was performed using the Flex Imaging 4.0 (Bruker Daltonics) software package. The correlation of the target plate with the optical image was performed from three distinct teaching points following the procedure of the Flex Imaging software (Bruker Daltonics).

## Additional Information

**How to cite this article**: Le Pogam, P. *et al.* Spatial mapping of lichen specialized metabolites using LDI-MSI: chemical ecology issues for *Ophioparma ventosa. Sci. Rep.* **6**, 37807; doi: 10.1038/srep37807 (2016).

**Publisher's note:** Springer Nature remains neutral with regard to jurisdictional claims in published maps and institutional affiliations.

## Supplementary Material

Supplementary Information
