# Supplementary material for: Spatial mapping of lichen specialized metabolites using LDI-MSI: chemical ecology issues for Ophioparma ventosa
Source: Sci Rep. 2016 Nov 24;6:37807. doi: 10.1038/srep37807 (PMC5121634; doi:10.1038/srep37807)
Supplement: Supplementary Information [file srep37807-s1.doc]

**Spatial mapping of lichen specialized metabolites using LDI-MSI: chemical ecology issues for *Ophioparma ventosa***

**Pierre Le Pogam**^1,*^**, Béatrice Legouin**^1^**, Audrey Geairon**^2^**, Hélène Rogniaux**^2^**, Françoise Lohézic-Le Dévéhat**^1^**, Walter Obermayer**^3^**, Joël Boustie**^1,*,+^**, and Anne-Cécile Le Lamer**^1,4,+^

^1^ Université Rennes 1, UMR CNRS 6226 PNSCM, 2 Avenue du Pr. L. Bernard, 35043 Rennes, ^2^ INRA UR 1268 BIA, Plate-forme BIBS, 44300 Nantes, ^3^ Universitat Graz, Institut Karl Franzens, Holteigasse 6, A-8010 Graz, ^4^ Université Toulouse 3 Paul Sabatier, UFR Pharmacie, 118 Route de Narbonne, 31062 Toulouse

* pierre.lepogam.alluard@gmail.com, joel.boustie@univ-rennes1.fr

+ these authors contributed equally to this work


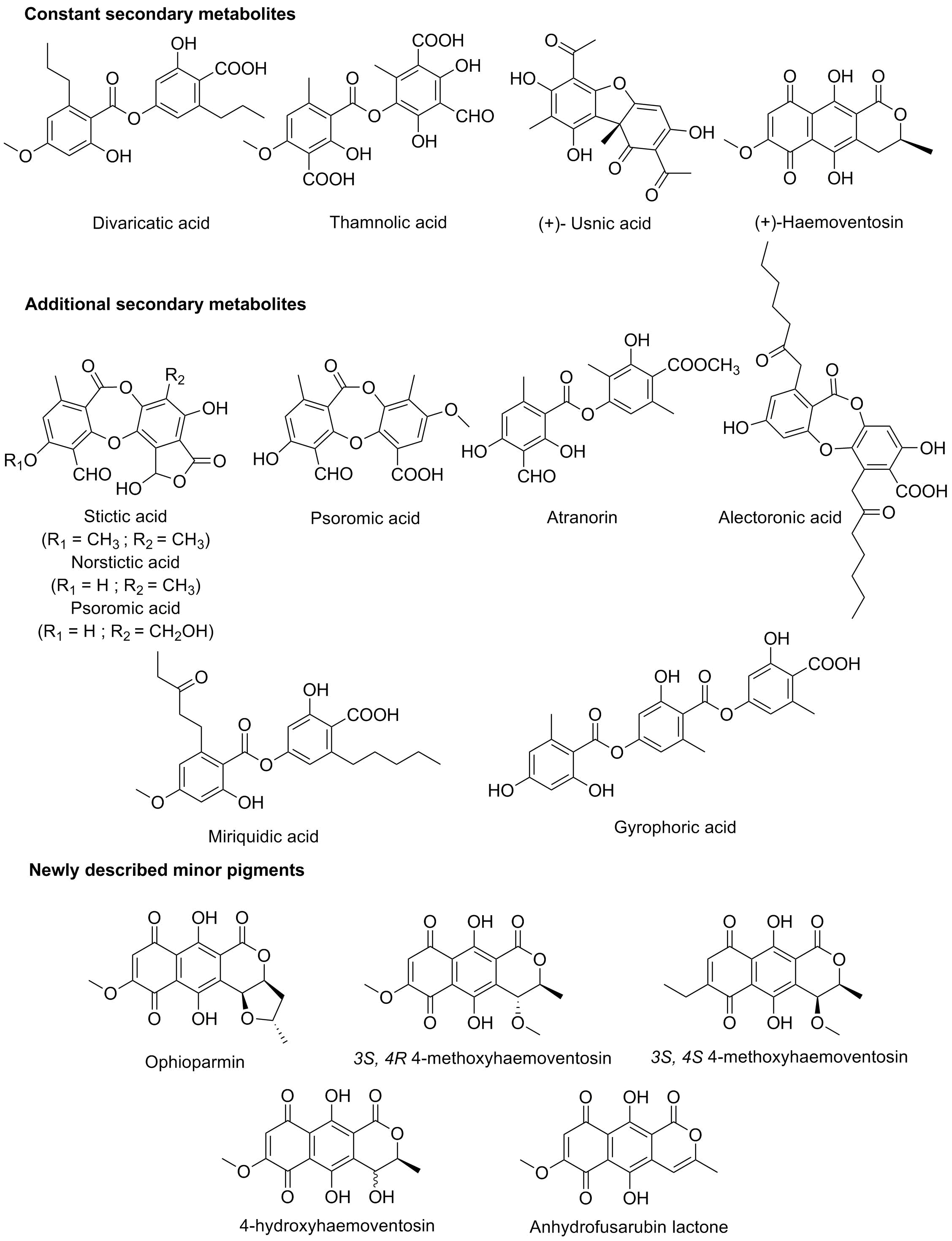


Figure S1 : Chemical structures of metabolites reported from Ophioparma ventosa

**Chemical investigation of microsamples of *Ophioparma ventosa* thallus**

**Experimental data**

Regarding the preparation of microsamples, random pieces or selected pieces of thallus (*ca*. 75 mg) were cut in the middle of the medulla using a razor blade prior to being crushed with mortar and pestle and subsequently extracted with 1 mL of dichloromethane for three hours. Extracts were then spotted on analytical TLC plates (Merck Silica Gel 60F254) using toluene/ethyl acetate/formic acid (70/25/5). Visualization of the plates was carried out under UV light (254 and 365 nm) and using anisaldehyde/H_2_SO_4_ reagent when heating.

**Preliminary localization of miriquidic acid on micro-samples**

The TLC monitoring of micro-samples’ dichloromethane extract revealed a distribution pattern comparable to that reported during our *in situ* DART-MS investigation, with usnic acid predominantly occurring in the upper layers and divaricatic acid evidenced in deeper strata. Representative examples obtained from the Styria sample are given in Fig. S2. One should note that usnic acid sometimes substantially arises within the lower half of the thallus.


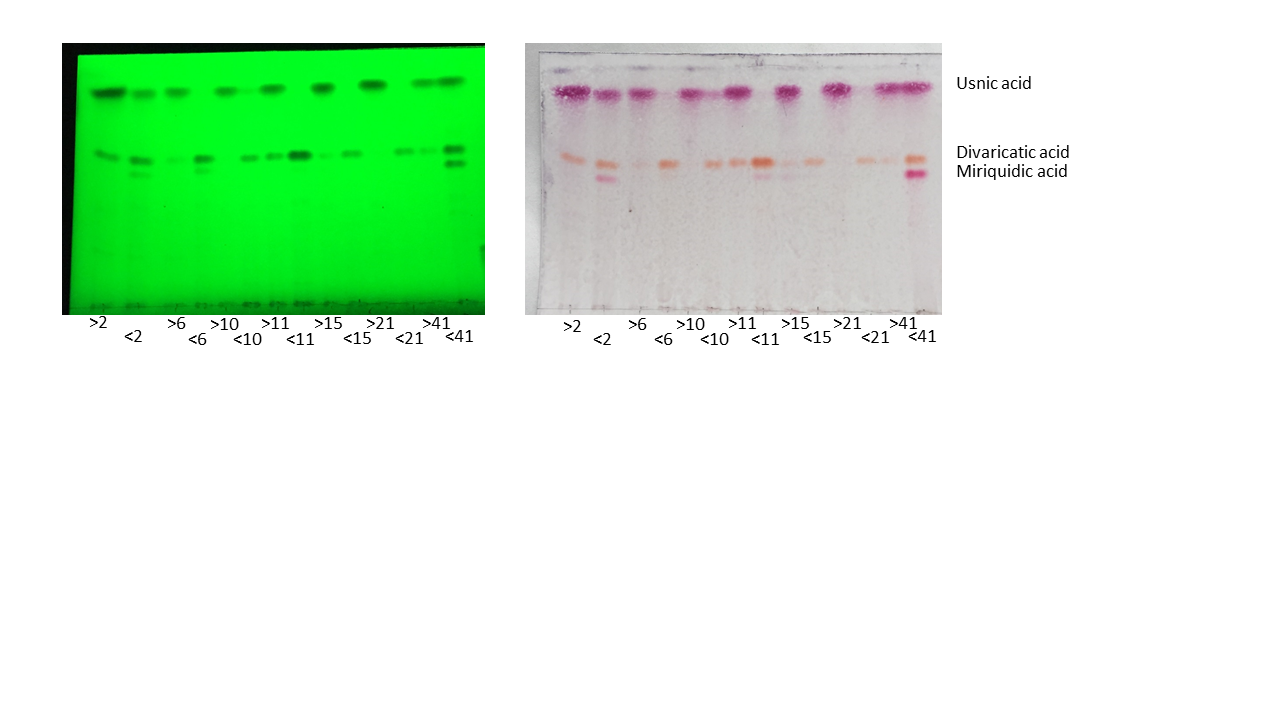


Figure S2: TLC of selected dichloromethane extract of upper and lower micro-samples of O. ventosa revealing the basal distribution of miriquidic acid and its uneven occurrence between different pieces of thallus. Left : UV 254 nm, right : plate sprayed using anisaldehyde


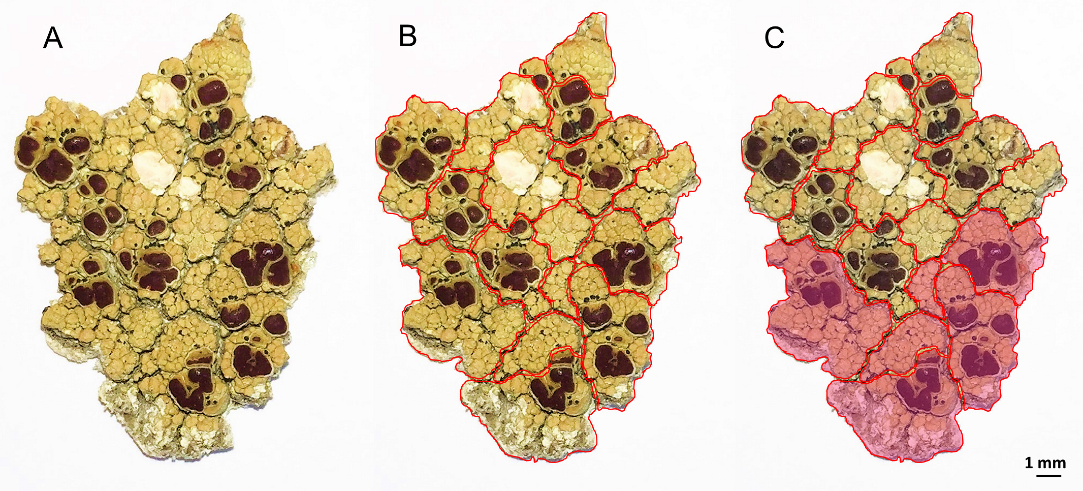


Figure S3: Longitudinal distribution of miriquidic acid in a piece of *Ophioparma ventosa* thallus (Tyrol sample). Division of a piece of thallus in small fragments (B). Rose patches refer to areas containing miriquidic acid (C)

**LDI-imaging of further slices**

The distribution pattern of all *Ophioparma ventosa* metabolites in slices lacking miriquidic acid is here presented on two supplementary sets of Figures.


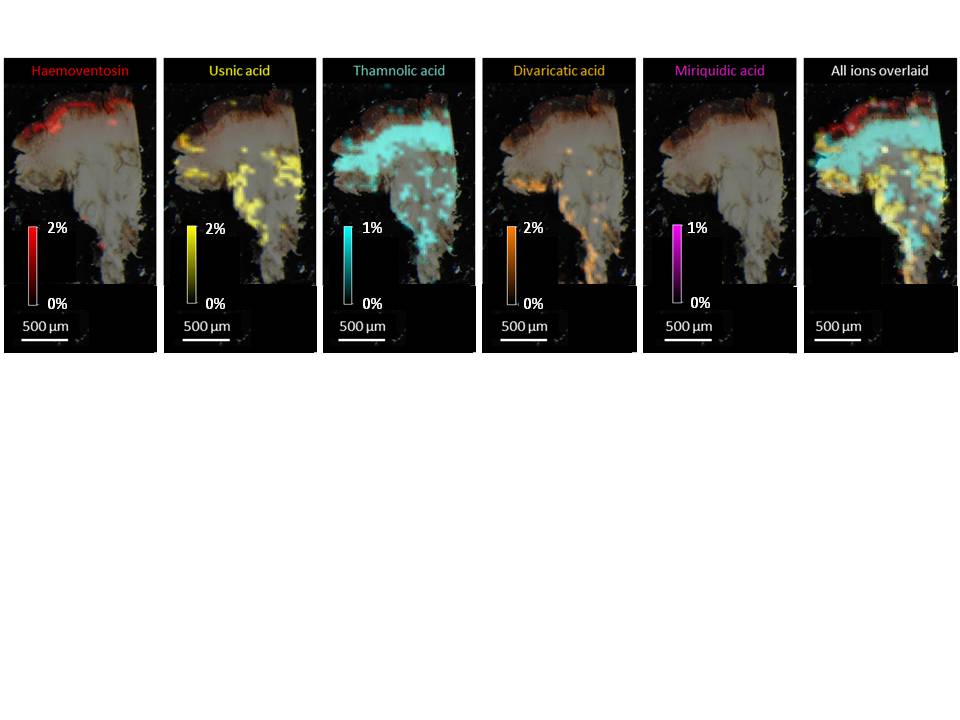


Figure S4: Spatial allocation of O. ventosa metabolites in a hand-cut apotheciate section obtained from the Tyrol sample. Intensity of ions of the imaged spots are color coded using a heat map with relative intensities given as indicated on the color scale bars.


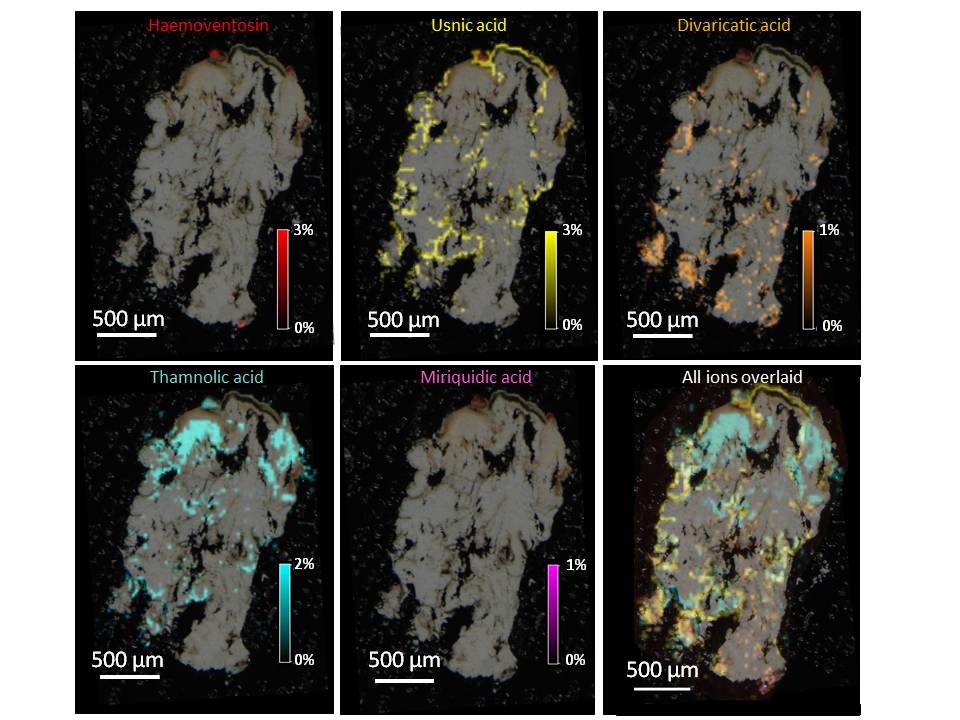


Figure S5: Distribution pattern of O. ventosa molecules in a cryosectioned slice from the Styria sample. Intensity of ions of the imaged spots are color coded using a heat map with relative intensities given as indicated on the color scale bars.
